# Optimizing accuracy: a comparative analysis of preoperative liver volumetry in living donor liver transplantation from a surgeon’s perspective – a retrospective cohort study

**DOI:** 10.1097/JS9.0000000000003112

**Published:** 2025-07-24

**Authors:** EunJin Choi, Seok-Hwan Kim

**Affiliations:** Department of Surgery, Chungnam National University College of Medicine, Daejeon, Korea

**Keywords:** dry weight correction factor, graft weight prediction, liver volumetry, living donor liver transplantation, retrospective cohort study, tertiary center

## Abstract

**Background:**

Accurate preoperative graft volume assessment is fundamental to the success of living donor liver transplantation (LDLT). Although manual and automated computed tomography (CT) volume measurement methods using various volumetric tools are widely used, their accuracy remains uncertain. This study aimed to compare various CT-based volumetric measurement methods for predicting actual graft weight (AGW) in LDLT and to identify specific dry weight correction factors for each method to improve clinical reliability.

**Materials and methods:**

A retrospective diagnostic accuracy study was performed on 109 patients who underwent LDLT between 2011 and 2024. Right liver volume was measured using automated (Philips Healthcare software), semi-automated (AnyVol software), and manual volumetry (PetaVision for clinics) methods. The optimal dry weight correction factor was calculated for each method.

**Results:**

The optimal dry weight correction factors were 0.89 for the automated method, 0.82 for the semi-automated method, and 0.88 for the manual method. After applying these correction factors, the semi-automated method yielded the highest coefficient of determination (*R*^2^ = 0.687, standard error [SE] = 91.939). The error ratio decreased significantly: from 11.30% ± 14.59% to −0.93% ± 12.98% for the automated method, from 20.51% ± 15.65% to −1.18% ± 12.83% for the semi-automated method, and from 11.89% ± 14.67% to −1.53% ± 12.91% for the manual method.

**Conclusions:**

Accurate prediction of AGW depends on applying optimal correction factors specific to each measurement method. All three methods showed high accuracy with the semi-automated method demonstrating the highest *R*^2^ and lowest SE, while the automated method exhibited the lowest error ratio. These findings support the use of cost-effective, software-based volumetry with tailored correction factors to improve donor safety and graft outcomes.

## Introduction

Living donor liver transplantation (LDLT) is a curative treatment for end-stage liver disease. Accurate preoperative assessment of the liver graft volume is essential for successful outcomes[1]. Ensuring donor safety requires the remnant liver volume to comprise at least 30%–40% of the total liver volume[2]. A graft recipient weight ratio (GRWR) of ≥0.8% is generally necessary to avoid small-for-size syndrome (SFSS)[3].

Various methods have been developed to obtain accurate liver volume measurements, with manually computed tomography (CT) volumetry being the most widely used approach in clinical practice[4]. However, manual CT volumetry is time-consuming[5] and labor-intensive, and its results largely depend on the observer’s precision. Advances in medical imaging and artificial intelligence (AI) have led to automated and semi-automated CT volumetry systems, offering potential reductions in time, labor, and variability^[6,7]^, thereby improving overall cost-efficiency in clinical practice. Despite these advancements, there is still debate regarding the method that provides more accurate measurements[8].

Significant differences were observed between the estimated graft volume (EGV) measured using CT volumetry and the actual graft weight (AGW) measured during LDLT. If the EGV overestimates graft weight, the actual graft may be insufficient for the recipient, potentially leading to graft failure. Conversely, if the EGV underestimates the graft weight, the donor’s remaining liver volume may be inadequate, heightening the risk of postoperative liver dysfunction. To ensure the safety of donors and recipients, it is imperative to minimize the differences between the EGV and AGW.

This study aimed primarily at addressing two key objectives: (1) To determine the optimal dry weight correction factors for automated, semi-automated, and manual CT volumetric methods, thereby improving the prediction accuracy of AGW in LDLT. (2) To systematically compare the accuracies of these three CT volumetric methods in predicting AGW.

## Materials and methods

This retrospective cohort study was performed with the approval of an institutional review board. The requirement for informed consent was waived. The study adhered to the ethical standards outlined in the Declaration of Helsinki and complied with relevant national and institutional guidelines. All data were anonymized to ensure participants’ confidentiality and privacy. This retrospective cohort study has been reported in line with the Strengthening the Reporting of Cohort, Cross-Sectional and Case-Control Studies in Surgery 2025 guidelines[9]. A systematic review of PubMed, Embase, and Google Scholar (up to October 2024) used terms like “liver transplantation,” “living donor,” and “volumetry.” No AI tools were used in data generation, analysis, or manuscript writing.

### Study population

**Figure 1.**
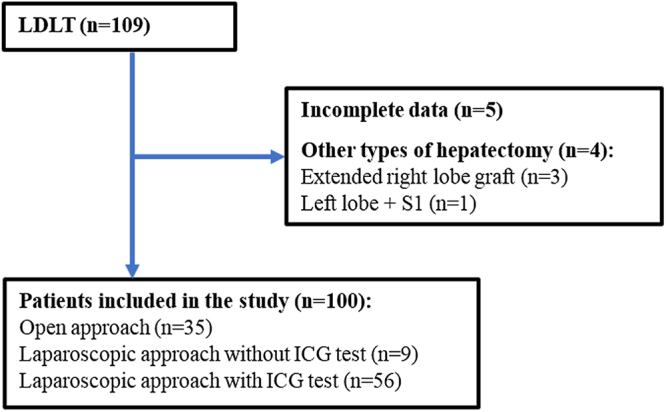
Flowchart of the study population.

Between January 2011 and December 2024, 109 LDLTs were performed at a tertiary care teaching hospital. We included all consecutive living donors who underwent right hepatectomy for LDLT and had complete preoperative CT volumetry data. Among these, five LDLT cases were excluded due to incomplete data, and four involving extended right lobe and left lobe grafts were excluded from the analysis. A total of 100 donors were included in the final analysis. Of these, 35 patients underwent open approaches, 9 underwent laparoscopic approaches without intraoperative indocyanine green (ICG) tests, and 56 underwent laparoscopic approaches with intraoperative ICG tests (Fig. 1). There were 66 male (66.0%), with a mean age of 30.10 ± 8.96 years. All donors had healthy livers, and none exhibited steatosis.

### Data collection

We retrospectively reviewed electronic medical records (EMRs) to extract data, including donor (age, sex, body mass index [BMI], intraoperative liver biopsy, preoperative fibroscan, operation method, types of graft, hospital days, and complications) and recipient (GRWR) data (Table 1).

**Table 1:** Patient demographics

Variables	Values	
Age (years)	30.10 ± 8.96 (16–57)	
Sex (n) (male:female)	66:34	
BMI (kg/m^2^)	24.60 ± 3.17 (17.67–33.29)	
Intraoperative liver biopsy	<5% in all donors (steatosis)	
Preoperative fibroscan	3.4 ± 0.5 kPa (2.5–4.9)	
Types of graft	Right hepatectomy	
Operation method (*n*) (%)	Open	35 (35.0)
	Laparoscopy with ICG test	56 (56.0)
	Laparoscopy without ICG test	9 (9.0)
Hospital days (days)	10.12 ± 5.04 (6–40)	
Complications (*n*) (%)	None	87 (87.0)
	Total complications *(Clavien-Dindo Grade)*	13 (13.0)
	Re-Operation *(IIIb)*	4 (4.0)
	Operative site fluid collection *(IIIa)*	5 (5.0)
	Pleural effusion *(IIIa)*	4 (4.0)
	Pneumothorax *(IIIa)*	1 (1.0)
	Biliary stricture *(IIIa)*	3 (3.0)
	Bile leak *(IIIa)*	3 (3.0)
GRWR (%)	1.24 ± 0.36 (0.63–2.88)	

BMI, body mass index; ICG, indocyanine green; GRWR, graft-recipient weight ratio.

Data are presented as mean ± standard deviation and frequency. Some patients experienced multiple complications simultaneously.

**HIGHLIGHTS**

- This retrospective cohort study compared automated, semi-automated, and manual computed tomography volumetry in living donor liver transplantation.
- Method-specific dry weight correction factors significantly improved graft weight prediction: 0.89 for automated, 0.82 for semi-automated, and 0.88 for manual volumetry.
- The semi-automated method showed the highest *R*^2^ (0.687), while the automated method had the lowest post-correction error ratio (–0.93%).
- After correction, all methods achieved mean error ratio <±2%, supporting clinical reliability.
- Applying tailored correction factors to cost-effective software-based volumetry enhances preoperative accuracy and supports safer outcomes for both donors and recipients, while highlighting the need to address inter-observer variability.

### CT imaging

All donors underwent preoperative CT angiography using a 128-slice multidetector CT scanner (SOMATOM Definition Edge, Siemens) that captured images with a 3 mm thickness. A contrast medium (Iopromide, Ultravist 300, Bayer) was administered intravenously at 2 mL/kg body weight, followed by a 30 mL saline flush. Scanning encompassed pre- and post-contrast phases, including the early arterial, portal venous, and delayed phases. Portal venous phase images were used for liver volume calculations using automated, semi-automated, and manual segmentation methods.

### CT volumetry

**Figure 2.**
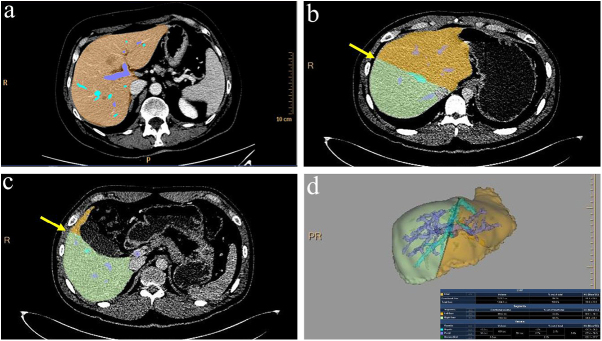
Automated volumetry of the liver by Philips Healthcare. (a) The liver was segmented on axial CT images, and the total liver volume was automatically computed. The system detected vascular structures, classifying them as portal, hepatic, or unclassified vessels, which were included in the volume analysis. (b) A radiologist defined the planned resection plane to the right of the middle hepatic vein (arrow). (c) The radiologist delineated the resection plane starting from the gallbladder bed (arrow). (d) The artificial intelligence (AI) algorithm automatically propagated the defined resection planes and mapped the major vascular structures to enhance surgical planning.

**Figure 3.**
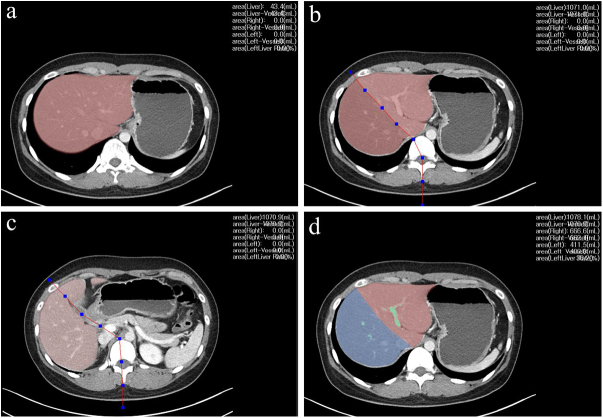
Semi-automated volumetry of the liver by AnyVol. (a) A surgeon manually traced the liver boundary on a single axial slice. The software completed the segmentation across subsequent slices and calculated the liver volume. (b) The surgeon established the resection plane to the right of the middle hepatic vein. (c) The surgeon outlined the planned dissection plane originating from the gallbladder bed. (d) Using AI-based processing, the system extended the surgeon-defined planes and detected the major vessels to refine the surgical strategy.

**Figure 4.**
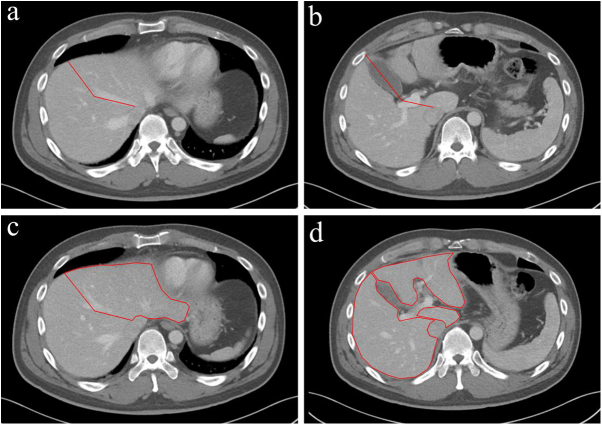
Manual volumetry of the liver by PetaVision. (a, b) A surgeon manually placed the dissection lines on all axial planes to delineate surgical boundaries. (c, d) For each axial image, the surgeon manually outlined the contours of the right liver volume, left liver volume, and segment 1 liver volume. The delineation process excluded the main vascular structures to ensure accurate volume measurements.

The CT liver analysis software (Philips Healthcare) was used for automated volumetry. The liver was identified on axial CT scans, and the total liver volume was calculated automatically. Manual adjustments to the liver boundaries were performed if necessary. Vessels were automatically detected, categorized as portal, hepatic, or unclassified, and included in the volume calculations. A radiologist placed two dissection lines – one to the right of the middle hepatic vein (MHV) and the other from the gallbladder (GB) bed – to define the right and left lobe volumes (Fig. 2). The time required for automated volumetry was measured as the total duration from the initiation of file upload to the completion of the automated process, including the radiologist’s input for adjustments and dissection lines. AnyVol, an open-source liver volumetry software, was used for semi-automated volumetric analysis^[4,10]^. The surgeon outlined the liver on one axial slice, and the software automatically delineated the remaining boundaries and calculated the liver volume. The software automatically identified vessels; however, only the main vessels were included in the volumetric calculations. Similar to the automated method, the surgeon marked two dissection lines: one to the right of the MHV and the other on the GB bed. The AI algorithm automatically generated the remaining dissection lines (Fig. 3). The time for semi-automated volumetry was similarly measured as the total duration from file upload to the completion of all processes, including the surgeon’s initial outline and dissection line adjustments. The primary PetaVision for clinics tool was used for the manual volumetry method^[11,12]^. The surgeon manually set the dissection lines on all axial planes, and outlined the contours of the right, left, and segment 1 liver volumes on each axial image, excluding the main vessels during the delineation process (Fig. 4). The time required for manual volumetry was calculated as the total duration from file upload initiation to the completion of all manual outlining processes, including all dissection line placements. To minimize bias, volumetric measurements were conducted in a blinded manner without access to the AGW data.

### AGW measurement after harvest procedure

To prevent fluid-related graft volume reduction, the AGW was measured intraoperatively immediately after graft procurement before flushing with a high osmolar preservation solution^[13,14]^. Blood was carefully and thoroughly removed from the graft through gentle manual compression and drainage of residual blood and fluid from hepatic veins. A calibrated electronic scale (accurate to the nearest gram) was used for precise measurement. This standardized protocol ensured accurate and reproducible graft weight measurements, providing a reliable benchmark for comparison with preoperative CT volumetry. The AGW was measured on the back table by an operator not involved in preoperative volumetry and blinded to the volume results to minimize measurement bias.

### Dry weight correction factor

To determine the optimal dry weight correction factor according to the automated, semi-automated, and manual CT volumetry methods, dry weight correction factors ranging from 0.8 to 0.95 at 0.01 intervals were applied to the graft volumes measured by each method. The differences between each corrected value and AGW were calculated by summing the absolute values of these differences. By comparing these sums across the different CT volumetric methods and correction factors, we identified the correction factor with the smallest total absolute difference for each method.

### Statistical analysis

Continuous variables are presented as mean and standard deviation, and all categorical variables are presented as frequencies and percentages.

Measurement accuracy was assessed using the coefficient of determination (*R*^2^), defined explicitly as the proportion of variance in AGW explained by EGV. Pearson’s correlation was used to demonstrate the correlation between AGW and EGV and was estimated using three volumetric methods. Correlation coefficients with 95% confidence intervals (CIs), full regression equations, and box plots illustrating error distribution and variability for each volumetric method were provided. Pearson’s correlation was also used in volume estimations to examine the correlation between donor characteristics (age and BMI) and the error ratio. The agreement between EGV and AGW was assessed using a modified Bland–Altman analysis, which calculates the mean difference and 95% limits of agreement. The reliability of the methods was evaluated using the intraclass correlation coefficient (ICC) with a two-way mixed-effects model.

The difference between the estimated right lobe and graft weight and error ratio[13] in volume estimation by each program was calculated using the following formulae:

$Difference\left(mL\right)=estimated\;\;right\;\;lobe-AGW$

$Error\;ratio\;\left(\%\right)=\left(estimated\;\;right\;\;lobe-AGW\right)/AGW\times 100\%$

A *P*-value <0.05 was considered statistically significant.

Repeated measures analysis of variance (ANOVA) was used to compare measurement times among the three methods. A univariate factorial ANOVA was conducted to identify statistically significant relationships between sex, operation methods, and error ratio in volume estimations. Statistical analyses were conducted using the SPSS software (version 26, IBM Corp.).

## Theory

In LDLT, accurate estimation of graft size is vital for donor safety and recipient viability. The two key metrics are EGV from preoperative imaging and AGW measured during surgery. While these values are expected to correlate closely, discrepancies can arise due to physiological and technical factors.

One significant factor is liver tissue density, which can vary based on age, steatosis, and vascular content. Additionally, variations in CT volumetry software’s segmentation boundaries, particularly around blood vessels, can lead to volume estimation errors.

To mitigate these issues, specific dry weight correction factors have been developed to adjust EGV for a better approximation of AGW. These factors vary by segmentation method, user interaction, and institutional protocols, highlighting the importance of evaluating and validating them to reduce the risk of graft mismatch.

## Results

### Patient characteristics

Of the 100 donors included in the final analysis, 66 were male, and 34 were female. The mean age of the donors was 30.10 ± 8.96 years (range, 16–57). The mean BMI was 24.60 ± 3.17 kg/m^2^ (17.67–33.29). Each donor underwent intraoperative liver biopsy, which demonstrated hepatic steatosis <5%. Preoperative liver stiffness measured by Fibroscan averaged 3.4 ± 0.5 kPa (range, 2.5–4.9). All donors underwent right hepatectomy, excluding the MHV. The mean hospital duration of the donors was 10.12 ± 5.04 days (range, 6–40). Complications were observed in 13 donors (13.0%), whereas 87 donors (87.0%) experienced no complications. Among those with complications, four donors (4.0%) required reoperation, including one for drain removal, one for adhesiolysis, and two for biliary tree exploration. Other complications included five cases (5.0%) of operative site fluid collection, four cases (4.0%) of pleural effusion, one case (1.0%) of pneumothorax, three cases (3.0%) of biliary stricture, and three cases (3.0%) of bile leakage. Notably, some patients experienced multiple simultaneous complications. The mean GRWR was 1.24% ± 0.36% (range, 0.63–2.88) (Table 1).

### The measured liver volume by volumetry

The mean estimated total liver volume was 1371.5 ± 293.6 mL for automated, 1459.9 ± 315.9 mL for semi-automated, and 1320.5 ± 274.3 mL for manual liver volumetry. The mean estimated right lobe volume was 865.3 ± 185.6 mL for automated, 939.9 ± 213.8 mL for semi-automated, 871.8 ± 193.7 mL for manual liver volumetry. The mean AGW was 783.7 ± 163.5 g.

### Dry weight correction factor calculation

The optimal correction factors were 0.89 for the automated, 0.82 for the semi-automated, and 0.88 for the manual methods, yielding total absolute differences of 7788.42, 7800.93, and 7458.18, respectively. Additional values are summarized in Table 2.

**Table 2:** Dry weight correction factor calculation (sum of absolute difference values)

Dry weight correction factor	Sum of absolute difference values		
	Automated	Semi-automated	Manual
0.8	10710.48	7939.48	10138.95
0.81	10172.87	7802.99	9580.47
0.82	9719.74	**7800.93**	9041.51
0.83	9301.97	7886.24	8594.65
0.84	8898.69	8075.80	8216.99
0.85	8551.52	8291.51	7895.96
0.86	8277.34	8519.33	7646.19
0.87	8048.71	8758.92	7495.91
0.88	7896.46	9068.84	**7458.18**
0.89	**7788.42**	9435.90	7465.82
0.9	7792.06	9867.57	7543.81
0.91	7919.31	10377.15	7698.10
0.92	8132.55	10934.19	7907.97
0.93	8375.88	11535.23	8187.19
0.94	8683.39	12177.23	8506.62
0.95	9042.81	12840.67	8882.01

Table 2 highlights the sum of absolute difference values for the dry weight correction factor across automated, semi-automated, and manual methods, with the automated method exhibiting consistently lower values.

### Association and agreement between EGV and AGW

**Figure 5.**
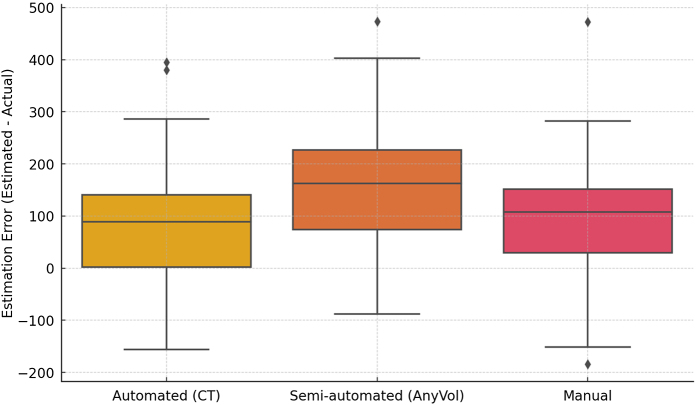
Distribution of estimation errors across methods. Estimation errors are shown for automated (CT-based), semi-automated (AnyVol), and manual segmentation methods. The central horizontal line in each box indicates the median error, while the box boundaries represent the interquartile range (IQR). Whiskers extend to 1.5× IQR, and outliers are indicated by diamonds. The automated method demonstrated lower median and interquartile estimation errors than the semi-automated and manual methods. These results visually support the quantitative findings showing method-specific performance in predicting actual graft weight.

Pearson’s correlation and linear regression analyses demonstrated strong associations between EGV and AGW across all methods: automated (*r* = 0.808, *R*^2^ = 0.652, 95% CI = 0.722–0.869), semi-automated (*r* = 0.829, *R*^2^ = 0.687, 95% CI = 0.751–0.884), and manual (*r* = 0.825, *R*^2^ = 0.680, 95% CI = 0.746–0.881). Although no significant differences were observed among the correlation coefficients of the three volumetry methods (*P* = 0.947, 0.691, and 0.740), each method demonstrated distinct performance characteristics. After applying the optimal dry weight correction factors (0.89 for automated, 0.82 for semi-automated, and 0.88 for manual), the semi-automated method achieved the highest coefficient of determination (*R*^2^ = 0.687, standard error [SE] = 91.939), indicating that it accounted for the greatest variance in AGW. The corresponding regression equations were AGW = 168.056 + 0.799 × EGV_automated, AGW = 187.877 + 0.634 × EGV_semi-automated, and AGW = 177.033 + 0.791 × EGV_manual. Box plots illustrated the distribution and variability of prediction errors across methods (Fig. 5).

**Figure 6.**
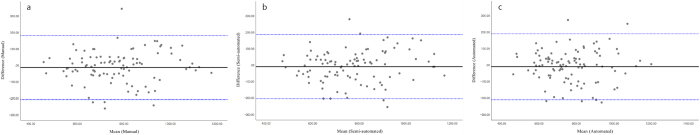
Modified Bland–Altman plots showing the agreement between EGV and AGW for the three volumetric methods. Plots are displayed for each method: automated (a), semi-automated (b), and manual (c). The solid line represents the mean difference (bias), and the dotted lines indicate the 95% limits of agreement.

By applying the 0.82 dry weight correction factor, the semi-automated method demonstrated the most potent predictive capability for AGW. Modified Bland–Altman analysis showed acceptable agreement for all methods, with mean differences of −13.54 g (automated), −12.95 g (semi-automated), and −16.49 g (manual) (Fig. 6). ICCs also demonstrated excellent agreement with values of 0.898, 0.909, and 0.906, respectively, supporting the reliability of each method (Table 3).

**Table 3:** Accuracy and reliability analyses of automated, semi-automated, and manual volumetric methods

Method	Coefficient of determination (*R*^2^)	mBland-Altman analysis(mean difference, MD)	ICC
Automated	0.652	MD: −13.54 g	0.898
		LOA: −213.32 to 186.24 g	95% CI: 0.847–0.930
Semi-automated	0.687	MD: −12.95 g	0.909
		LOA: −208.38 to 182.47 g	95% CI: 0.861–0.941
Manual	0.680	MD: −16.49 g	0.906
		LOA: −210.74–177.77 g	95% CI: 0.854–0.941

mBland-Altman analysis, modified Bland-Altman analysis; ICC, intraclass correlation coefficient; LOA, limits of agreement; CI, confidence interval.

Mean volumetric ratios were consistent across methods – 63.3% (automated), 64.4% (semi-automated), and 65.9% (manual) – and remained stable after AGW adjustment, supporting their reliability for donor safety evaluation.

### Comparisons of error ratio

**Figure 7.**
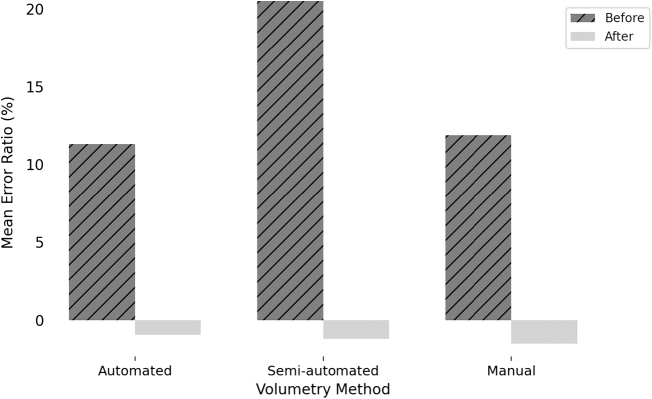
Error ratio comparison before and after applying dry weight correction factor. Before correction, the mean error ratio was 11.30% ± 14.59% (automated), 20.51% ± 15.65% (semi-automated), and 11.89 ± 14.67% (manual). After correction, the automated method showed the lowest mean error ratio (−0.93% ± 12.98%), followed by the semi-automated (−1.18% ± 12.83%) and manual methods (−1.53% ± 12.91%).

As shown in Figure 7, the mean error ratio was 11.30% ± 14.59% for the automated method, 20.51% ± 15.65% for the semi-automated method, and 11.89% ± 14.67% for the manual method before applying the dry weight correction factor. After applying the optimal dry weight correction factor for each method, the automated method exhibited the lowest mean error ratio, −0.93% ± 12.98%, followed by the semi-automated method, −1.18% ± 12.83%, and the manual method, −1.53% ± 12.91% (Fig. 7). The error ratio analysis suggests that, when adjusted with the appropriate dry weight correction factor, automated volumetry provides the most accurate estimation of AGW among the methods evaluated.

### Comparisons of measurement times

The mean measurement times were 4.38 ± 1.24 min for the automated method, 21.91 ± 4.14 min for the semi-automated method, and 30.20 ± 6.28 min for the manual method. A multivariate analysis revealed significant differences among the three methods [Wilks’ λ = 0.029, *F*(2, 98) = 1636.267, *P* < 0.001], with a large effect size (partial *η*^2^ = 0.971). Owing to sphericity violation (Mauchly’s *W* = 0.643, *P* < 0.001), Greenhouse-Geisser corrections were applied. Repeated measures ANOVA confirmed significant differences among the methods [*F*(1.474, 145.878) = 861.075, *P* < 0.001; partial *η*^2^ = 0.897]. Bonferroni-adjusted pairwise comparisons indicated significant differences between all methods (*P* < 0.001). A linear trend was observed, with measurement times progressively increasing from the automated to semi-automated and manual methods [*F*(1, 99) = 1646.134, *P* < 0.001].

### Analysis of the relationship between patient characteristics and error ratio

Correlation analysis examined the relationships among age, BMI, and error ratios of the three volumetric methods. There was no significant correlation between age and the error ratio (automated: *r* = 0.101, *P* = 0.341; semi-automated: *r* = 0.122, *P* = 0.251; manual: *r* = 0.097, *P* = 0.363). Similarly, BMI showed no significant correlation with the error ratios (automated: *r* = −0.111, *P* = 0.297; semi-automated: *r* = −0.022, *P* = 0.838; manual: *r* = −0.123, *P* = 0.245).

The error ratios for the three volumetric methods were compared based on sex and operative methods using one-way ANOVA, revealing no significant difference in the error ratio of the automated method based on sex (*F* = 0.002, *P* = 0.961). Similarly, for the semi-automated and manual volumetric methods, the mean error ratios for males and females did not differ significantly (semi-automated: *F* = 2.014, *P* = 0.159; manual: *F* = 1.896, *P* = 0.172). When comparing error ratios by surgical methods (open, laparoscopic ICG-, laparoscopic ICG+), no statistically significant differences were observed for all three volumetric methods (automated: *F* = 2.594, *P* = 0.080; semi-automated: *F* = 2.221, *P* = 0.115; manual: *F* = 1.720, *P* = 0.185).

## Discussion

A graft dry weight correction factor was used to account for the differences between the EGV and AGW. Hwang *et al*[15] measured the amount of blood in a living donor liver graft and analyzed the correlation between the volumetric graft volume and graft weight, arriving at a correction factor of 0.82. Karlo *et al*[16] suggested a dry weight correction factor of 0.85 for CT volumetry. Notably, the dry weight correction factor varies within a range of 0.8–0.95 across different studies, reflecting the variability in methodologies and patient populations. Given that these variabilities are usually due to the volumetric method used, a tailored approach for each measurement technique is necessary to achieve more accurate graft volume assessment and improve the clinical outcomes of LDLT.

We identified a discrepancy between the EGV and AGW before applying the optimal dry weight correction factor. Several factors may account for this variance. First, the difference between the expected and actual dissection lines may be a significant factor. There is no guarantee that the resection lines determined preoperatively using CT scans will be followed precisely during surgery^[17,18]^. Additionally, in preoperative auto- or semi-auto-CT volumetric methods, a radiologist or surgeon defines the initial dissection lines only on the first and second images. The AI automatically generates the remaining slides, leading to these discrepancies. Marcos *et al*[19] reported that a 2 cm difference between radiological and actual dissection lines could result in a 200 g variance in graft weight. Second, a fundamental difference exists between liver conditions during CT imaging and immediately after harvest. During preoperative volumetric CT imaging, the liver is in a physiological condition and perfused with blood at normal physiological levels, and injection of a contrast agent can also influence the fluid status^[20,21]^. When AGW measurements are performed immediately after harvest, even in the nonperfused state, a large amount of blood is removed, resulting in a notable decrease in the liver volume compared to the normal physiological state[15]. Although large blood vessels are excluded from CT volumetry calculations, this method is inaccurate for estimating the amount of blood in the liver[22]. Third, although commonly used, the assumption of a 1:1 conversion ratio between liver volume and graft weight remains controversial. Heinemann *et al*[23] calculated an average tissue density of 1.08 g/mL between liver volume and weight in 33 healthy livers using the Archimedes principle. Van Thiel *et al*[24] reported a correlation of 0.99 between liver volume and weight in cirrhotic livers. Moreover, the liver density may increase if the liver undergoes steatosis[25]. A small-scale study involving 16 live liver donors found that the average hepatic density was roughly 12% higher than 1.00 g/mL, with significant individual variations in density[26]. Furthermore, a small tissue sample obtained from a liver biopsy may not accurately reflect the overall condition of the entire right liver graft when assessing the degree of fatty liver. Consequently, discrepancies are inevitable.

Even after applying the optimal dry weight correction factor, there were cases in which the error ratio exceeded the 10% deviation range for all three methods. To identify the potential causes of these discrepancies, we hypothesized that the variations in volume status between the time of CT imaging and surgery might contribute to the differences between the EGV and AGW. To assess this, we measured the diameter ratio of the inferior vena cava (IVC), as its flatness indicates the volume status[27]. The IVC diameter ratio was measured on axial images obtained during the portal venous phase of preoperative CT scans. Specifically, the IVC diameter ratio was defined as the ratio of the maximum transverse diameter (side-to-side width) to the maximum anteroposterior diameter (front-to-back depth), measured at the infrarenal level immediately below the confluence of the renal veins[28]. Our analysis revealed no statistically significant correlation between the IVC diameter ratio and error ratio across all three methods: automated (*r* = −0.325, *P* = 0.121), semi-automated (*r* = −0.286, *P* = 0.175), and manual (*r* = −0.344, *P* = 0.100). This outcome suggests that variations in the volume status did not substantially influence the observed discrepancies.

The GRWR is a critical parameter in LDLT, as a GRWR below 0.8% increases the risk of SFSS and graft failure[3]. Our study data identified four cases in which the preoperatively estimated GRWR was above 0.8%, whereas the actual GRWR was much lower postoperatively. When applying the optimal dry weight correction factor to calculate the EGV, three of the four cases were predicted to have a GRWR <0.8%, allowing for a more accurate preoperative prediction of GRWR. Specifically, in two cases where the postoperative GRWR was 0.63% and 0.68%, the EGV-predicted GRWR was 0.81% and 0.84%, respectively. This adjustment suggests that using the EGV adjusted with the optimal dry weight correction factor to calculate the GRWR can reliably ensure the safety threshold for liver transplant recipients.

An error >10% in graft weight prediction can affect outcomes like SFSS[29]. In this study, all methods achieved mean errors within ±2% after correction, supporting their clinical reliability for graft weight estimation in LDLT.

In our study, the semi-automated method demonstrated the highest *R*^2^, while the automated method showed the lowest error ratio post-correction. These differences suggest that the automated method may be better suited for precise individual predictions, whereas the semi-automated method offers stronger trend-level consistency. While prior studies (e.g. Kalshabay *et al*[8]) favored manual and automated methods, these differences may reflect variations in software, workflows, and our use of surgeon-guided inputs in the semi-automated platform.

We used a Philips CT scanner and an automated volumetry program compatible with this widely adopted platform. For semi-automated analysis, we employed AnyVol, a free open-source software that demonstrated the highest accuracy among the three methods. Both automated and semi-automated programs are easily accessible and cost-effective, leading to significant reductions in time, cost, and labor, especially when compared to high-end three-dimensional reconstruction platforms, which require greater financial and infrastructural resources. The automated method offers speed and is suitable for rapid assessments, while the semi-automated method, though slower, provides greater predictive consistency (highest *R*^2^). The manual process is accurate but resource-intensive and best reserved for specialized cases.

By applying the optimal dry weight correction factor proposed in this study, these software tools can offer highly accurate measurements of liver volume in LDLT, potentially enhancing the outcomes for donors and recipients. Method selection should be guided by clinical context, balancing precision, efficiency, and resource availability.

Our study has some limitations. First, this study utilized retrospective data collected from EMR, which inherently carries the potential for information bias. To address this, we carefully included only cases with complete and reliable data, ensuring robust analysis. Second, we exclusively analyzed right lobe grafts, the predominant graft type in adult LDLT^[30–32]^. Other graft types – including extended right lobe, left lobe, and left lateral segment grafts commonly used in pediatric transplantation – may exhibit different EGV–AGW discrepancies due to anatomical and vascular variations; however, their limited use at our center precluded meaningful comparative analysis. As such, our findings may not be generalizable to these graft types, and further validation through multicenter studies involving diverse graft types is warranted. Third, a significant limitation is observer variability, as different raters performed different methods. To address this, future studies should assess inter-observer reliability using multiple raters per method to quantify variability better and strengthen methodological rigor.

Additionally, their applicability to other centers remains uncertain because the correction factors were derived from a single-center cohort using institution-specific volumetry tools and protocols. External validation in multicenter prospective studies is warranted.

## Conclusions

Accurate measurement of liver volume and prediction of liver graft weight are foundational to ensuring donor safety and recipient outcomes in LDLT. This study calculated the optimal dry weight correction factors for different measurement methods: 0.89 for automated, 0.82 for semi-automated (using AnyVol), and 0.88 for manual methods, respectively. The application of these factors significantly reduced the error ratio. The semi-automated method exhibited the highest *R*^2^ and lowest SE, whereas the automated method exhibited the lowest error ratio. These findings highlight the importance of applying method-specific correction factors to improve the reliability of liver-volume assessments. Combining readily available, cost-effective software tools with optimal dry weight correction factors can enhance preoperative planning, ensure safer donor outcomes, and improve graft function in recipients.

Future studies should address these limitations, validate these findings across other graft types, and further explore inter-observer differences to refine volumetric methods and improve LDLT outcomes.

## Data Availability

This study was registered at ClinicalTrials.gov under the registration number NCT06865612, where the full study protocol is available. The datasets generated and analyzed during the current study are available from the corresponding author upon reasonable request.

## Research registration unique identifying number (UIN)

This study was registered at ClinicalTrials.gov under the registration number NCT06865612, where the full study protocol is available.

## Assistance with the study

The authors would like to thank the other HBP staff of Chungnam National University Hospital for their invaluable support throughout the study. The authors would like to thank Professor Je-Hui Seo for their valuable statistical consultation and support in the data analysis of this study. The authors would like to thank Editage (www.editage.com) for English language editing.
